# 3D Neuronal Cell Culture Modeling Based on Highly Porous Ultra-High Molecular Weight Polyethylene

**DOI:** 10.3390/molecules27072087

**Published:** 2022-03-24

**Authors:** Aleksey A. Ustyugov, Nataliya A. Sipyagina, Alena N. Malkova, Elena A. Straumal, Lyudmila L. Yurkova, Anastasiya A. Globa, Maria A. Lapshina, Maria M. Chicheva, Kirill D. Chaprov, Aleksey V. Maksimkin, Sergey A. Lermontov

**Affiliations:** 1 Institute of Physiologically Active Compounds of the Russian Academy of Sciences, 1 Severnij proezd, 142432 Chernogolovka, Russia; dolmatin_89@mail.ru (N.A.S.); amalkova81@gmail.com (A.N.M.); lenochka.chg@gmail.com (E.A.S.); ludmilaush@mail.ru (L.L.Y.); globa271194@mail.ru (A.A.G.); lapshina.masha@yandex.ru (M.A.L.); chicheva.mariya@gmail.com (M.M.C.); chapkir@gmail.com (K.D.C.); aleksey_maksimkin@mail.ru (A.V.M.); lermontov52@yandex.ru (S.A.L.); 2 Center of Composite Material, National University of Science and Technology “MISIS”, Leninsky pr. 4, 119049 Moscow, Russia

**Keywords:** UHMWPE, 3D cell culture, scaffolds, cell function

## Abstract

Cell culturing methods in its classical 2D approach have limitations associated with altered cell morphology, gene expression patterns, migration, cell cycle and proliferation. Moreover, high throughput drug screening is mainly performed on 2D cell cultures which are physiologically far from proper cell functions resulting in inadequate hit-compounds which subsequently fail. A shift to 3D culturing protocols could solve issues with altered cell biochemistry and signaling which would lead to a proper recapitulation of physiological conditions in test systems. Here, we examined porous ultra-high molecular weight polyethylene (UHMWPE) as an inexpensive and robust material with varying pore sizes for cell culturing. We tested and developed culturing protocols for immortalized human neuroblastoma and primary mice hippocampal cells which resulted in high rate of cell penetration within one week of cultivation. UHMWPE was additionally functionalized with gelatin, poly-L-lysine, BSA and chitosan, resulting in increased cell penetrations of the material. We have also successfully traced GFP-tagged cells which were grown on a UHMWPE sample after one week from implantation into mice brain. Our findings highlight the importance of UHMWPE use as a 3D matrix and show new possibilities arising from the use of cheap and chemically homogeneous material for studying various types of cell-surface interactions further improving cell adhesion, viability and biocompatibility.

## 1. Introduction

Three-dimensional cell culturing techniques are gaining momentum in recent years. The main drive for the development is based on the hypothesis that most cell types and particularly neuronal cells require a 3D environment for proper functioning. The brain itself could be viewed in this regard as a complicated matrix which encompasses proper structural components needed for cell bodies to be implanted (“grey matter”) with the myelinated axons comprising the “white matter”. Numerous studies have already shown that cells grown in 3D conditions as compared to a conventional 2D monolayer are altered in morphology and gene expression patterns [1,2], migration [3] as well as cell cycle and proliferation [4]. In 2D conditions, cells have to adapt to a flat and hard surface resulting in altered cellular metabolism, biochemical features, cell cycle kinetics, morphology, functionality and cellular and physiological responses compared to a native whole organ condition. For that matter, the use of 3D neuronal culture reduces the extent of limitations attributed to in vitro cell models especially when it comes to the selection of potential drugs [5]. Therefore, 3D cellular models are much more relevant from a biological perspective and represent a new direction and a modern trend in biological research.

The cellular matrix material must be biocompatible/bioinert yet provide support for the formation of a life system with open interconnected pores to ensure the appropriate livable conditions—supply of nutrients, removal of waste products as well as creating an environment for intercellular communication. There are numerous scaffold-based materials which are used for cell growth as substrates mimicking extracellular matrix. These matrixes are referred to as hydrogels and solid scaffolds with various origins [6,7]. Yet, a limited number of materials are available specifically for use as a neuronal scaffold. Three-dimensional-printed material based on graphene and polylactide-co-glycolide was used to cultivate neuronal cells with directional conductivity from pluripotent cells and was tested in the operative treatment of spine injury of rats [8]. There is evidence of unstructured 3D cellular models created by directional cell differentiation from hiPSCs in a growth medium in the absence of a structuring agent and substances that promote adhesion. As a result, structures called human cortical spheroids are obtained which could be used to model processes occurring in neuronal tissues [9].

The bulk of modern research in the field of 3D cultivation of neuronal tissue is devoted to the development of new composite hydrogels based on protein components with the addition of synthetic structures that promote directed growth and differentiation of cultures according to the neuronal type. This is achieved through the deposition of growth factors in the gel. It was recently shown that a hydrogel based on the self-organizing peptide MAX8 forms a network with the necessary pore sizes for cell culture growth under physiological conditions. The experiments were performed using human medulloblastoma cells, which is a tumor that occurs in the cerebellum in childhood [10]. However, these studies have revealed the main disadvantage of using hydrogels—the appearance of necrotic areas. These areas are formed due to hindered outflow of waste products as well as limited access to nutrient media. This issue was partially resolved by creating a 3D printed material based on multilayered carbon nanotubes built into the structure of the PEGDA polymer. The material has high electrical conductivity which allows the neuronal cell culture to maintain its functionality attributed to nerve tissue [11]. As a result, an alternative material aiming at generation of 3D neuronal cultures on carbon nanotubes (CNTs) was proposed. The material was prepared by mixing modified CNTs with a chitin solution and showed excellent properties in culturing of PC12 and RSC96 cells with improved proliferative indices and dendritic networks [12]. However, the toxic effect of CNTs use for growing neuronal cultures was also revealed. Other composite scaffolds based on CNT were also proposed. The addition of hyaluronic acid as the base for the hydrogel showed neural differentiation of human embryonic neural stem cells [13]. Another study showed a hybrid hydrogel containing graphene to support the growth of cultured brain cells facilitating synaptic activity [14] as well as a new type of hydrogel called micro-TENN stimulated the longitudinal growth of neurons along the hydrogel strand which could be possibly used in regenerative medicine in spinal cord injuries [15].

Despite being a preferred choice, hydrogel with the above-mentioned modifications is quite cumbersome to produce and involves resource-consuming steps which could hinder scaling up production. Therefore, ultra-high molecular weight polyethylene (UHMWPE) could serve as a promising material for 3D matrix. UHMWPE represents a polymer with a molecular weight of over 106 Da. It is inexpensive, hailed for its bioinert properties, durability and capability to form porous structures. In addition, it has high biocompatibility and biostability, chemical inertness, lubricity, impact resistance and abrasion resistance [16,17,18,19,20].

It was shown that porous UHMWPE has a structure mimicking cancellous tissue, did not cause cytotoxicity and hemolysis, provided effective tissue ingrowth and could be applied in reconstructive surgery for replacement of non-loaded parts of bones [21]. Subcutaneous implantation of porous UHMWPE scaffolds in mice demonstrated material–tissue interaction, development of granulation tissue, fibrosis and fibrous capsule development [17,22]. The cells were actively attached to the scaffold with the subsequent granulation and vascularization of the synthetic scaffold with no negative tissue reaction to the implant in vivo. Porous UHMWPE scaffold containing hydroxyapatite caused effective bone growth which was shown in a model of the skull bones in mice, and the combination of UHMWPE scaffold with BMP-2 protein led to intensive mineralization of new bone tissue as early as three weeks after implantation [23].

In this work, a porous UHMWPE prepared by salt leaching (pores ~50–900 µm) was used as a cell matrix to culture primary neuronal and human neuroblastoma cells. Our findings suggest that UHMWPE is a versatile material with excellent structural properties and can be used as an inexpensive and robust matrix to study 3D cell–cell interactions and neuronal cell physiology

## 2. Results and Discussion

**Figure 1:**
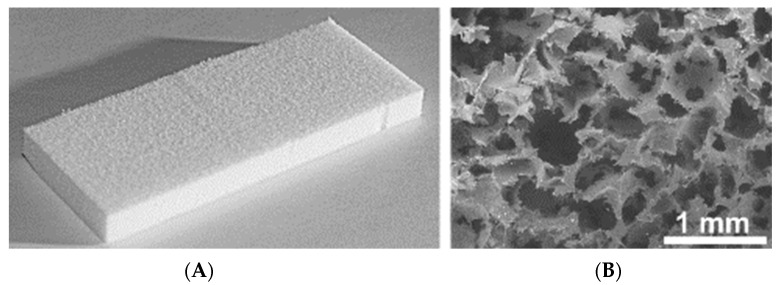
Image (**A**) and Scanning Electron Microscopy micrograph (**B**) of the porous UHMWPE sample.

Figure 1A and Scanning Electron Microscopy micrograph of an untreated UHMWPE sample with a porous structure exhibits open interconnected large macropores, mostly spherical in shape (Figure 1). Macropores range in size from 100 to 800 microns, which is determined by the size of the salt particles used in UHMWPE preparation. The surface of large macropores has a microrelief with some roughness caused by contact with the surface of NaCl particles.

### 2.1. Growth of SH-SY5Y Cells and Primary Hippocampal Cell Cultures on UHMWPE Matrix

**Figure 2:**
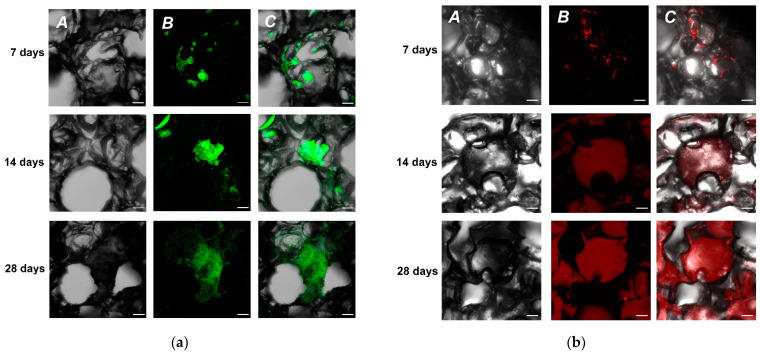
Confocal images of cell growth of SH-SY5Y-F15 and primary mice hippocampal culture on UHMWPE matrix at 7, 14 and 28 days. (**a**) SH-SY5Y-F15 cells: panel A—3D porous structure of UHMWPE, phase contrast (grey); panel B—SH-SY5Y constitutively expressing GFP (green); (**b**) primary culture cells: panel A—3D porous structure of UHMWPE, phase contrast (grey); panel B—primary hippocampal culture stained with Rhodamine-phalloidin (red). Panel C on (**a**,**b**)—merged images of panels A and B. Scale—100 µm.

We have examined two different cell culture types to test our initial hypothesis, whether it was possible to grow cells in UHMWPE. We used immortalized neuroblastoma SH-SY5Y cells constitutively expressing green fluorescence protein (GFP) as a marker. This cell line was readily available and did not require any specific modifications for fast and robust cell visualizations. Since it was an in-house derived modified cell line, we called it SH-SY5Y-F15. Currently, our cell screening assays for selection of promising neuroprotective agents also involve primary hippocampal cultures. Thus, in parallel to SH-SY5Y-F15, we used hippocampal primary cell culture from P1 mice pups as a source material for seeding UHMWPE. As a result, we showed that using confocal microscopy it was possible to detect cells on the third day of cultivation for both cell types (Supplementary Materials, Figure S1). It was also found that many pores with complex geometry were formed inside the material, which apparently aided cells to successfully penetrate deep into the sample. On day seven, the cells formed complex highly organized structures up to 500 µm in size, filling the free pores (Figure 2).

It is worth noting that in a traditional 2D format at the same initial seeding density that was used for the UHMWPE samples, a monolayer is formed. This monolayer hinders further cell growth and proliferation. Yet, UHMWPE culturing continued and by the 14th day of cultivation, cells occupied from 50 to 70% of the surface of the UHMWPE. It was evident that with a timely change of medium, neuroblastoma cells were capable of further growth. As a result, the experiment with SH-SY5Y-F15 was eventually aborted at day 60 (data not shown), since it was obvious that further growth of the cell culture was directly dependent on the availability of fresh nutrient medium with a UHMWPE sample cell density approaching 90%.

Rhodamine-phalloidin staining showed the absence of stress fibrils, which may indicate the stability of the cell cytoskeleton. The cells formed intercellular contacts, which indicate their viability and functional stability and integrity of the culture. Nuclear staining with DAPI also indicated the absence of DNA defects and nuclear integrity (data not shown), hence suggesting that the cell culture grown on UHMWPE has all the vital functions for stable growth. As a result, we concluded that both types of cultures could be actively grown on UHMWPE samples for prolonged duration of time compared to the traditional 2D conditions. Thus, UHMWPE can be used as a 3D matrix for long-term cultivation of cells without loss of their functional morphological features.

### 2.2. Analysis of the GFAP in Neuroblastoma SH-SY5Y-F15 Cells and Primary Hippocampal Culture on UHMWPE

**Figure 3:**
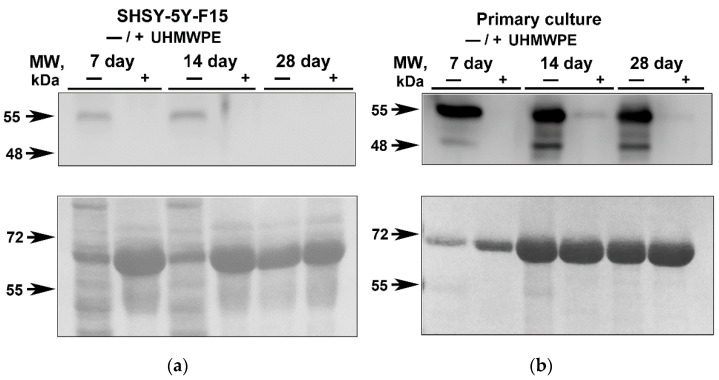
Detection of cytoplasmic GFAP level in cells seeded in 2D (−) compared to UHMWPE (+). (**a**) WB result for GFAP after 7, 14 and 28 days of human neuroblastoma cells (clone SHSY-5Y-F15); (**b**) WB result for GFAP after 7, 14 and 28 days in primary hippocampal cell cultures.

Glial fibrillar acidic protein (GFAP) is a structural component of the cytoskeleton in mature astrocytes, and it is often used as a neuronal marker and as an indirect marker of neuroinflammation. Typically, astrocytes delimitate damaged areas of the brain from undamaged structures and release proinflammatory factors activating inflammation as well as take part in the development of glial scars at the site of damage. Culturing cells in traditional 2D environment resulted in GFAP increase both for SH-SY5Y and primary hippocampal cultures [24]. At the same time, the production of GFAP in cells grown on UHMWPE is much lower (Figure 3). GFAP redistribution may indicate that UHMWPE culturing could reduce changes in cell structure and integrity leading to cell death or inflammatory reactions compared to the conventional culturing 2D methods (on glass/plate). However, confirmation of this hypothesis requires a more in-depth study, including detailed analysis of the expression levels of anti-inflammatory genes as well as more sophisticated techniques using inflammatory inhibitors. A key outcome of our work was the confirmation that growing cells in a 3D environment such as on UHMWPE matrices has a decreased GFAP production compared to the conventional 2D setups suggesting that 3D culturing conditions are closer to the physiological environment.

### 2.3. Obtaining Samples of Porous UHMWPE with Increased Surface Hydrophilicity. UHMWPE Functionalization with Biopolymers for Increased Biocompatibility

**Figure 4:**
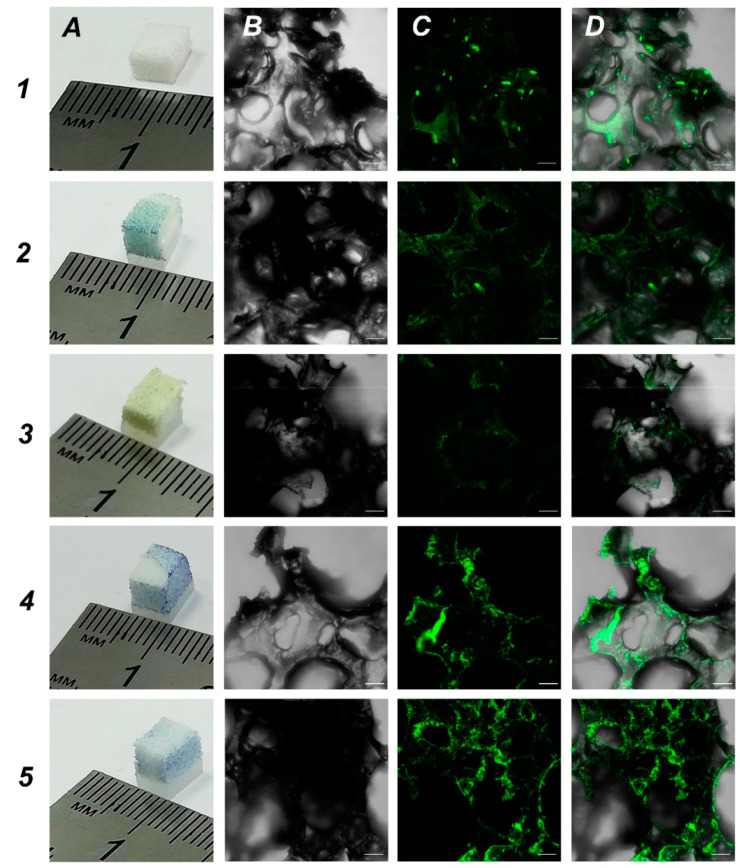
Visualization of SH-SY5Y-F15 cell growth on UHMWPE treated with various biopolymer compounds. Rows: 1—unmodified UHMWPE, 2—0.1% gelatin, 3—0.01% poly-L-lysine, 4—0.1% BSA, 5—0.1% chitosan. Panels: (**A**)—macro photographs of non-treated and treated UHMWPE; (**B**)—phase-contrast imaging (grey); (**C**)—SH-SY5Y cell expressing GFP (green); (**D**)—merged images (**B**) and (**C**). Scales for (**B**–**D**)—100 μm.

One of the main aims was to develop new 3D scaffolds suitable for growing cells and one of the tasks was to increase the hydrophilicity of UHMWPE for better cell adhesion. In order to obtain UHMWPE scaffolds with an increased hydrophilicity, we soaked UHMWPE in different biopolymer solutions. We used bromophenol blue as colorimetric dye to assess the functionalization of UHMWPE samples. We focused on biopolymers that are used for coating in cell culturing: 0.1% gelatin, 0.01% poly-L-lysine, 0.1% bovine serum albumin and 0.1% chitosan. These are the typical concentrations used in conventional 2D cell cultures. Based on the data obtained, all tested biopolymers penetrated UHMWPE samples which was visualized by a bromophenol blue dye (Figure 4, panel A). Then, we used our in-house SH-SY5Y-F15 cell line as a robust and available cell culture to evaluate biopolymer coated UHMWPE scaffolds. Our analysis showed that treatment with bovine serum albumin and chitosan were most effective on cell culture growth (Figure 4, rows 4 and 5, respectively). We believe that functionalization with biopolymers changed the topology in UHMWPE matrices leading to the most suitable parameters for cell growth.

### 2.4. UHMWPE as a Vehicle for Cell Delivery

**Figure 5:**
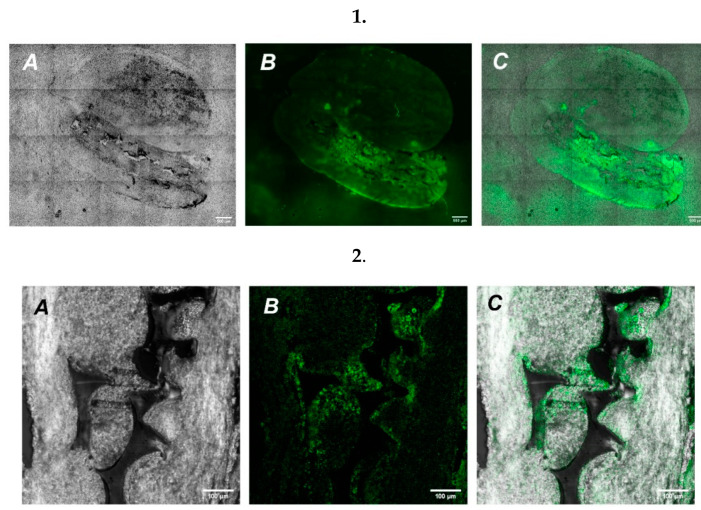
A series of confocal tilescans (Row **1**) and magnified high resolution images (Row **2**) of mice brain (frontal cut) at the 7th day after UHMWPE implantation. (**A**)—UHMWPE in brain tissue, phase contrast (grey); (**B**)—SH-SY5Y cells expressing GFP (green); (**C**)—merged. Scale: Row 1—500 µm, Row 2—100 µm.

To study the possibility of UHMWPE to serve as a vehicle for targeted cell delivery, we grew GFP expressing cells on UHMWPE and implanted a cultured UHMWPE cell-containing sample into the brains of mice. After implantation, the mice were closely monitored with the assessment of stress and overall physiological conditions. After a week-long recovery period, mice were terminally euthanized followed by brain extraction for further histological analysis. Microscopic examination of histological preparations of mouse brain slices revealed the site of UHMWPE scaffold implantation. Using high magnification on a confocal laser scanning microscope, we detected the implanted material containing GFP signal from the SH-SY5Y cells (Figure 5).

**Figure 6:**
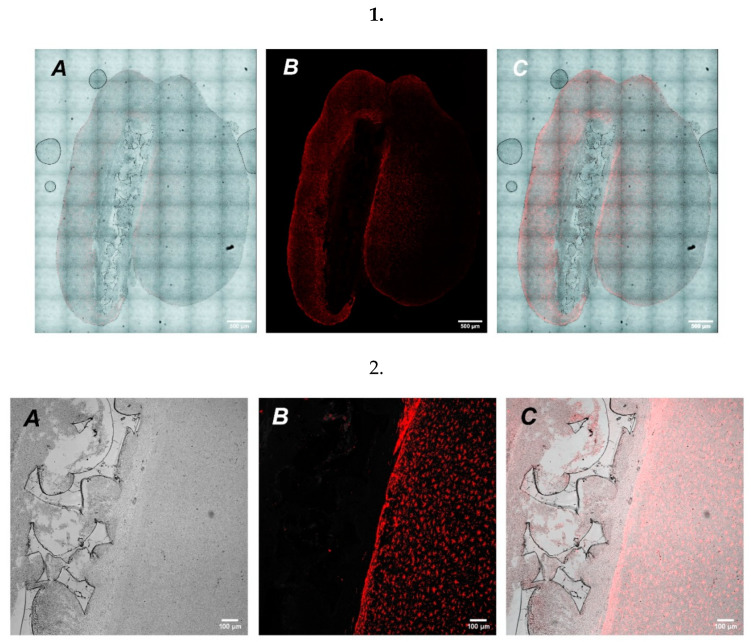
A series of confocal tilescans (Row **1**) and magnified high resolution images (Row **2**) of mice brain (frontal cut) at the 7th day after UHMWPE implantation. (**A**)—UHMWPE in brain tissue, phase contrast (grey); (**B**)—GFAP stained SH-SY5Y (red); (**C**)—merged. Scale: Row 1—500 µm, Row 2—100 µm.

In addition, we also analyzed whether implantation of cell-free UHMWPE samples could increase inflammatory response in mouse brains. We used GFAP as a marker for astrogliosis and found that there was a predominant increase in the periphery of the lesion (Figure 6), which could indicate restriction of the inflammation zone and the beginning of regeneration of the damaged nerve tissue at the site of UHMWPE implantation. The implication is that UHMWPE was successfully implanted into the brains of mice and could be potentially used as a cell carrier; however, more studies are needed to clarify the molecular aspects that are associated with the potency and safety of UHMWPE use as a delivery vehicle.

## 3. Conclusions

One of the primary outcomes was the proof of concept that UHMWPE is suited for culturing cells. This is the first study demonstrating that primary hippocampal neurons can be successfully cultured on UHMWPE. This is clearly of a great importance as it opens up wide opportunities for creating novel 3D cell models based on primary cultures. Ultimately, any transgenic animals, e.g., modeling various aspects of pathological conditions, could serve as a source for ex vivo 3D models. In turn, these novel 3D cell models could be extremely useful to study specific pathological events occurring in a 3D environment, complex cell–cell interactions, biochemical reactions and, most importantly, recapitulating controllable organ-like cell culture conditions. Without a doubt, these new 3D models based on UHMWPE step forward in generation of adequate cell systems for drug selection. In our study, we also developed microscopy techniques for fast and robust visualization of cells cultured in UHMWPE. The primary advantage of using UHMWPE was the fact that it is an inexpensive and easy to use material which could be easily shaped into a desired configuration depending on the cell culturing protocol. In addition, we have successfully functionalized UHMWPE with biopolymers including gelatin, poly-L-lysine, BSA and chitosan, with the last two yielding higher cell penetrations of the material.

Our findings highlight the importance of UHMWPE use and show the ability of new material for studying various types of cell-surface interactions of UHMWPE and functionalization parameters, further improving cell adhesion, viability and biocompatibility. Other future directions of UHMWPE use as a cell scaffold could involve cell reactors or inexpensive multilayer culturing setups. The advantage of using UHMWPE lies in the high surface-to-volume ratio yielding a material with widely branched surfaces of various sized pores which is not possible to achieve in other materials. The undoubted breakthrough was a UHMWPE implantation into mouse brains for the first time. Our findings are in consensus with other studies which use UHMWPE for various biological applications ranging from membrane to vascular stent graft applications [22,25,26]. Moreover, there is a growing body of studies suggesting UHWMPE as a promising polymer material for biomedical applications. In such a case, our study demonstrates the potential viability for UHWMPE as implantable scaffolds either carrying necessary cell types to target areas, such as in wound healing applications, or as a structural element for various medical aspects from cartilage replacement to strengthening blood vessels.

## 4. Materials and Methods

UHMWPE—GUR 4120 (Ticona) 5 × 10^6^ Da was used as received. An initial salt-leached sample was prepared as previously described [16,21,23,27]. UHMWPE and NaCl (with a particle size of 80–900 μm) composite powder (1:9 by weight) was obtained by mixing in a planetary ball mill (Fritsch Pulverisette 5) at low energy conditions. The final composite powder UHMWPE/NaCl was hot pressed at 180 °C and 70 MPa to obtain monolithic samples. NaCl was etched from UHMWPE matrix by boiling in distilled water. Water was removed by drying at 70 °C for 3 h.

The microstructure of the samples was studied using a Vega3 Tescan scanning electron microscope (SEM).

### 4.1. Antibodies

Anti-GFAP, rabbit polyclonal to GFAP (Abcam, ab7260) was used as a primary antibody to detect glial fibrillar acidic protein in Western blotting and immunohistochemical analysis, peroxidase AffiniPure Donkey Anti-Rabbit IgG (H + L) (Jackson ImmunoResearch Laboratories, 711-035-152) was used as a secondary antibody in Western blotting, Rhodamine (TRITC)-conjugated AffiniPure Donkey Anti-Rabbit IgG (H + L) (Jackson ImmunoResearch Laboratories, 711-025-152) was used as a secondary antibody in immunohistochemical analysis.

### 4.2. Cell Culture

#### 4.2.1. Primary Culture

The primary cell culture was prepared from the hippocampus of P1 mice. Isolated hippocampi were placed in a sterile Phosphate buffered saline pH 7.4 (PBS) solution of extra pure grade with the salt composition of 137 mM NaCl, 2.7 mM KCl. Then, the tissue samples were transferred into a 0.05% Trypsin solution and incubated at 37 °C for 15–20 min. Thereafter, the trypsinized samples were centrifuged at 2000 rpm for 5 min. The trypsin solution was decanted and 4 mL of full Neurobasal medium (Neurobasal 50 mL, pen/strep 833 μL, l-glu 125 μL, b-27 supplement 1 mL) was added, resuspended and re-centrifuged at 2000 rpm for 5 min. The medium was replaced, and the cell pellet was slowly resuspended. The prepared cell suspension was centrifuged at 2000 rpm for 5 min, the supernatant was discarded and the final cell suspension was prepared for seeding by adding full Neurobasal medium (1 part of the medium to 2 parts of the primary cell culture). Subsequently, upon seeding and cell adhesion, the medium was changed once a week in such a way that 50% of old medium remained.

#### 4.2.2. SH-SY5Y Human Neuroblastoma Cell Culture

SH-SY5Y-F15 modified human neuroblastoma cells (ATCC, CRL2266) constituently expressing green fluorescent protein (eGFP) were cultured in Dulbecco’s Modified Eagle Medium/Ham’s F-12 (DMEM/F-12) supplemented with 10% fetal bovine serum, 2 mM L-glutamine, 100 U/mL penicillin, 100 μg/mL of streptomycin and 2.5 μg/mL of amphotericin. Cells were cultured at 37 °C in a humidified atmosphere of air and 5% CO_2_ with changing medium twice a week.

### 4.3. 2D and 3D Culturing of Primary Culture Cells and Human Neuroblastoma

Two-dimensional culturing protocols for primary neuronal cells used 12 mm (size 2) glass coverslips treated with poly-L-lysine solution (Sigma, P4707). To recreate a 2D environment, SHSY5Y-F15 (5 × 10^5^ cells) per well or primary culture were plated onto coverslips in 24-well plates. The 3D culturing on UHMWPE was performed with the same cell suspension that was used in 2D culturing with a total of 100 μL/well of cells was pre-incubated for 2 h at 37 °C with the subsequent addition of 650 μL of full culture medium. The neuroblastoma culturing media was fully changed twice a week. For primary cultures, culturing media was changed once a week with 50–60% of the medium in the wells replaced with fresh nutrient medium. Cells were seeded onto UHMWPE slabs (2 × 4 × 6 mm) which were steam sterilized (110 °C, 1.2 atm., in a steam autoclave for 10 min) in a 24-well culture dish. One day prior to seeding, UHMWPE slabs were pre-soaked in culturing media to promote cell penetration upon seeding

### 4.4. Immunofluorescence Microscopy

Cells grown on coverslips and UHMWPE slabs were washed with PBS and fixed with 4% paraformaldehyde (PFA) solution at room temperature for 20 min and washed with PBS. Permeabilization was carried out with a 0.1% Triton-X 100 solution for 10 min, washing with PBS solution followed by overnight incubation at 4 °C with primary antibody solution. The next morning, cells were washed with a 0.05% Tween solution and incubated with secondary antibodies conjugated with Rhodamine phalloidin (TRITC) (R415, Invitrogen, Thermo Fisher Scientific, Waltham, MA, USA) for 2 h at room temperature. Cells were washed with 0.05% Tween solution and counterstained with DAPI solution (500 nM) for 10 min followed by 3 washes with PBS solution with subsequent mounting on glass slides. Fluorescent images were obtained using a Zeiss laser scanning microscope LSM 880 (Jena, Germany).

### 4.5. Protein Extraction from Cells

Cells growing on glass coverslips were collected by scraping and pelleted in protein collection sample buffer (25 mM Tris-HCl pH 7.6, 150 mM NaCl, 1% NP-40 (Nonidet P-40), 1% sodium deoxycholate, 0.1% SDS complemented with protease inhibitor cocktail), while cells growing on UHMWPE slabs were treated directly with sample buffer to collect protein extracts. Samples were incubated for 30 min at 4 °C followed by centrifugation for 10 min at 12,000 rpm. The supernatant was taken for protein electrophoresis in PAGE and immunoblotting by adding standard 2× Laemmli buffer.

### 4.6. Immunoblotting

SDS-PAGE proteins were transferred from the gels onto a Hybond-C Extra nitrocellulose membrane (Amercham) in a transfer buffer (25 mM Tris-HCl, 19.3 mM glycine, 20% methanol) at +4 °C and 100 mA for 2 h. The membrane was stained in Ponceau’s dye solution for 5 min to verify transfer and incubated in a blocking solution—TBST buffer (100 mM Tris-HCl, pH 7.5, 150 mM NaCl, 0.1% Tween-20) containing 5% bovine serum albumin (BSA), 5 fraction (VWR Life Science, cat # 0332-100G) and 0.02% NaN_3_ for 1 h at room temperature. Primary rabbit polyclonal antibody to GFAP (1:1000) was applied overnight at 4 °C on orbital shaker followed by washes next morning and a subsequent secondary HRP-conjugated antibody incubation (1:10,000) at room temperature in a blocking solution without NaN_3_ and washed three times with TBST buffer. The chemiluminescence reaction was carried out using a mixture of ECL solutions: 140 μL of solution A (0.68 mM p-coumaric acid in DMSO) and 10 μL of 30% H_2_O_2_ were added to 14 mL of solution B (100 mM Tris-HCl, pH 8.5, 1.25 mM luminol). The results of immunoblotting were detected using a UVP Bioimaging system (Upland, CA, USA).

### 4.7. Implantation of UHMWPE into Mouse Cortex

Mice were obtained from the Center for Collective Use of the Institute of Physiologically Active Compounds RAS (Chernogolovka, Russian Federation). Up to the ages of one and four months, the mice were housed in groups of five per cage; thereafter, each of them was kept for 1.5 months in an individual cage. Mice were housed in a standard environment (12-h light/dark cycle, 18–26 °C room temperatures and 30–70% relative humidity) with food and water ad libitum. The procedures were carried out in accordance with the “Guidelines for accommodation and care of animals. Species-specific provisions for laboratory rodents and rabbits” (GOST 33216-2014) and were in compliance with the principles enunciated in the Directive 2010/63/EU on the protection of animals used for scientific purposes and were approved by the local Institute of Physiologically Active Compounds Ethics Review Committee (protocol #30, 30 April 2019). All efforts were made to minimize the number of animals and their suffering. UHMWPE slabs were cultured with SHSY5Y-F15 cells prior to implantation. For anesthesia induction, an animal was placed in plexiglass chamber (150 × 100 × 100 mm, RWD Life Science Co., San Diego, CA, USA) under 4% isoflurane (Isoflurane, Laboratorios Karizoo, S.A., Barcelona, Spain) until the animal was fully anesthetized. UHMWPE implantation was performed in a stereotactic head frame (David Kopf Instruments, Germany) under constant 2% isoflurane active anesthesia (R500IE RWD Life Science Co., USA). Head was shaved of fur and cleaned with EtOH before incision. After skin 1 cm incision and removal of all soft tissue from the surface of the skull, a 5 mm hole was drilled (RWD Life Science Co., USA). The stereotaxic coordinates were established according to the mouse brain atlas and UHMWPE slabs were implanted bilaterally into primary motor cortices (M1) and putamen (Pt) (AP: +1.1 mm anterior to bregma; ML: ±1.5 mm lateral to midline; DV: −0.75 and −2.75 mm depths from skull surface, respectively) (Supplemental Figure S2) [28]. Following surgery, animals were individually housed with food and water available ad libitum. At least one week needed for recovery before terminal euthanasia and tissue collection.

### 4.8. Histology and Immunohistochemistry

For histological examination, the brains of mice were fixed in Carnoy’s solution (60% ethanol, 30% chloroform, 10% glacial acetic acid) at +4 °C for 16 h followed by dehydration using ethanol-chloroform and embedded in paraffin blocks [29,30]. The paraffin blocks were then cut using a Leica RM 2265 microtome (Leica Biosystems Inc., Deer Park, IL, USA). Serial sections (8 μm) were mounted on poly-L-lysine coated Leica X-tra Adhesive glasses (Leica Biosystems Inc., Deer Park, IL, USA). Then, the slides were subjected to immunohistochemical staining [31]. Brain sections were stained for astrocyte activation marker using a primary antibody against GFAP (polyclonal, rabbit anti-GFAP antibody, Abcam ab7260, dilution 1:300) and a secondary antibody with Alexa Fluor 568 (polyclonal, goat anti-rabbit IgG (H + L), Invitrogen A-11011, dilution 1:500). Brain sections were visualized on confocal laser scanning microscope (ZEISS LSM 880, Jena, Germany).

### 4.9. UHMWPE Functionalization

To obtain UHMWPE with increased hydrophilicity, samples of UHMWPE were soaked in various biopolymers. A dye (bromophenol blue) (Sigma B0126, St. Louis, MO, USA) was used to visualize the presence of biopolymer and then visualize the sample after implantation into the mouse brain. UHMWPE samples were soaked in 0.1% solution of gelatin (Sigma G1890, St. Louis, MO, USA) or bovine serum albumin (BSA Amresco 0332-100G, Lot #0198B3328, Solo, Newport News, VA, USA) and 0.01% solution of poly-L-lysine (PLL Sigma P-4832, St. Louis, MO, USA) dissolved in DI water or 0.1% solution of chitosan (Acros Organics, Geel, Belgium) dissolved in 1% CH_3_COOH/H_2_O followed by ultrasonic bath incubation for 20 min, and then left at room temperature for 24 h (air drying). The concentrations of selected biopolymers were based on their applications in conventional 2D culture protocols.

### 4.10. MTT Viability Assay

To investigate the viability of SHSY-5Y-F15 cells, the 3-(4,5-dimethylthiazol-2yl)-2,5-diphenyl-2H-tetrazoliumbromide (MTT) assay was performed [32]. At the end of the for 28 days of cell growth, the cell culture medium was replaced with fresh medium containing 0.5 mg/mL of (MTT) and the cells were incubated for 4 h. Then, culture medium was aspirated, and MTT-formazan-stained cells were imaged under light microscopy.

## Data Availability

Not applicable.

## Supplementary Materials

The following supporting information can be downloaded at: https://www.mdpi.com/article/10.3390/molecules27072087/s1, Figure S1: MTT/formazan colorimetric assay depicting single cells and cell colonies viability inside UHMWPE matrix. Figure S2: Illustration of UHMWPE implantation into mouse cortex.

## Sample Availability

Samples of the compounds are not available from the authors.

## Abbreviations

BSA	bovine serum albumin
CNTs	carbon nanotubes
DI	deionized
GFAP	glial fibrillar acidic protein
UHMWPE	Ultra-High Molecular Weight Polyethylene
