# Supplementary material for: 3D Neuronal Cell Culture Modeling Based on Highly Porous Ultra-High Molecular Weight Polyethylene
Source: Molecules. 2022 Mar 24;27(7):2087. doi: 10.3390/molecules27072087 (PMC9000589; doi:10.3390/molecules27072087)
Supplement: Supplementary file 1 [file molecules-27-02087-s001.zip › molecules-1599860-supplementary.pdf]

Supplemental Figure S1

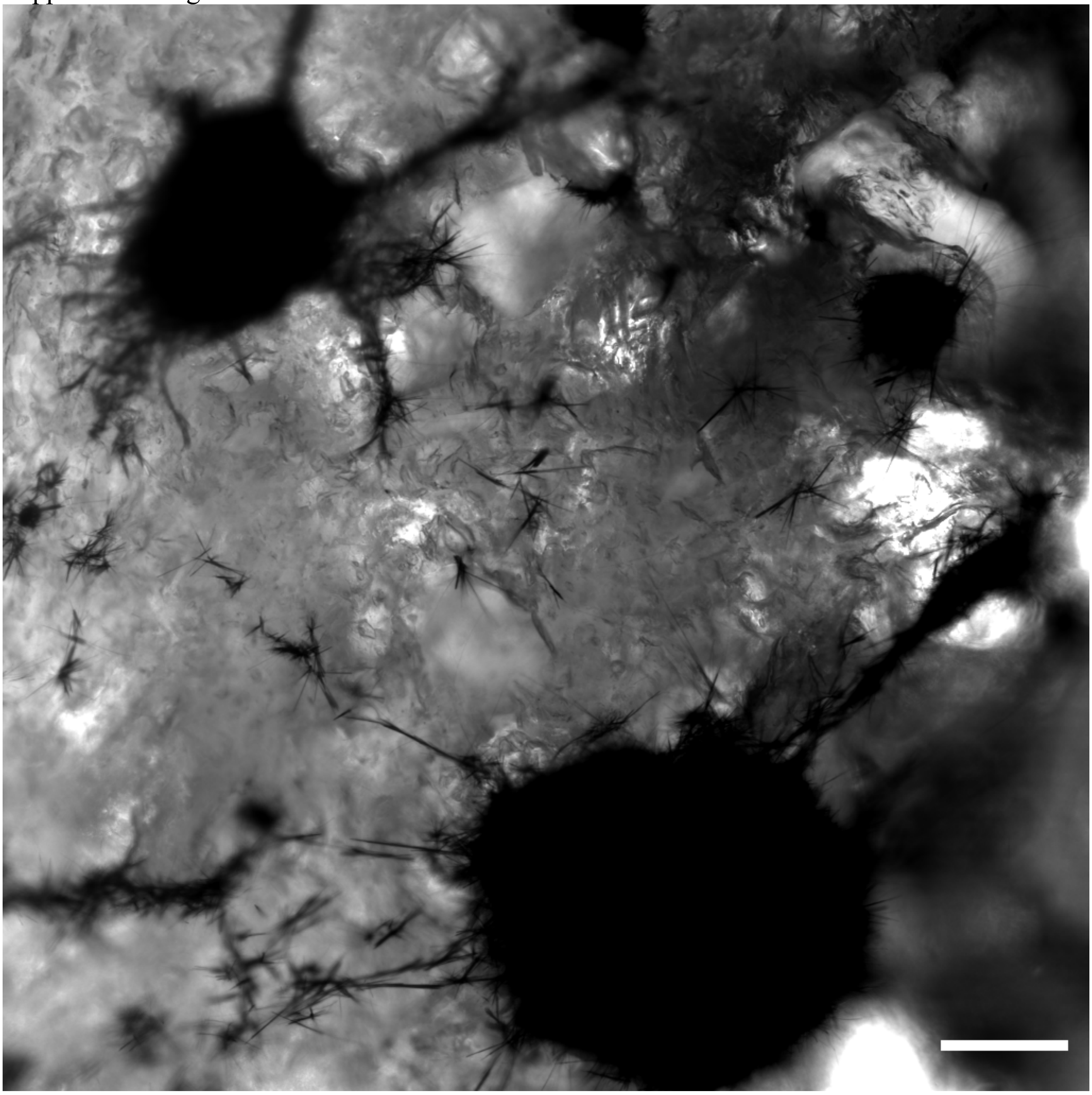

Supplemental Figure S1.
MTT/formazan colorimetric assay depicting single cells and cell colonies viability inside UHMWPE matrix. Scale bar 100 μm.

Supplemental Figure S2

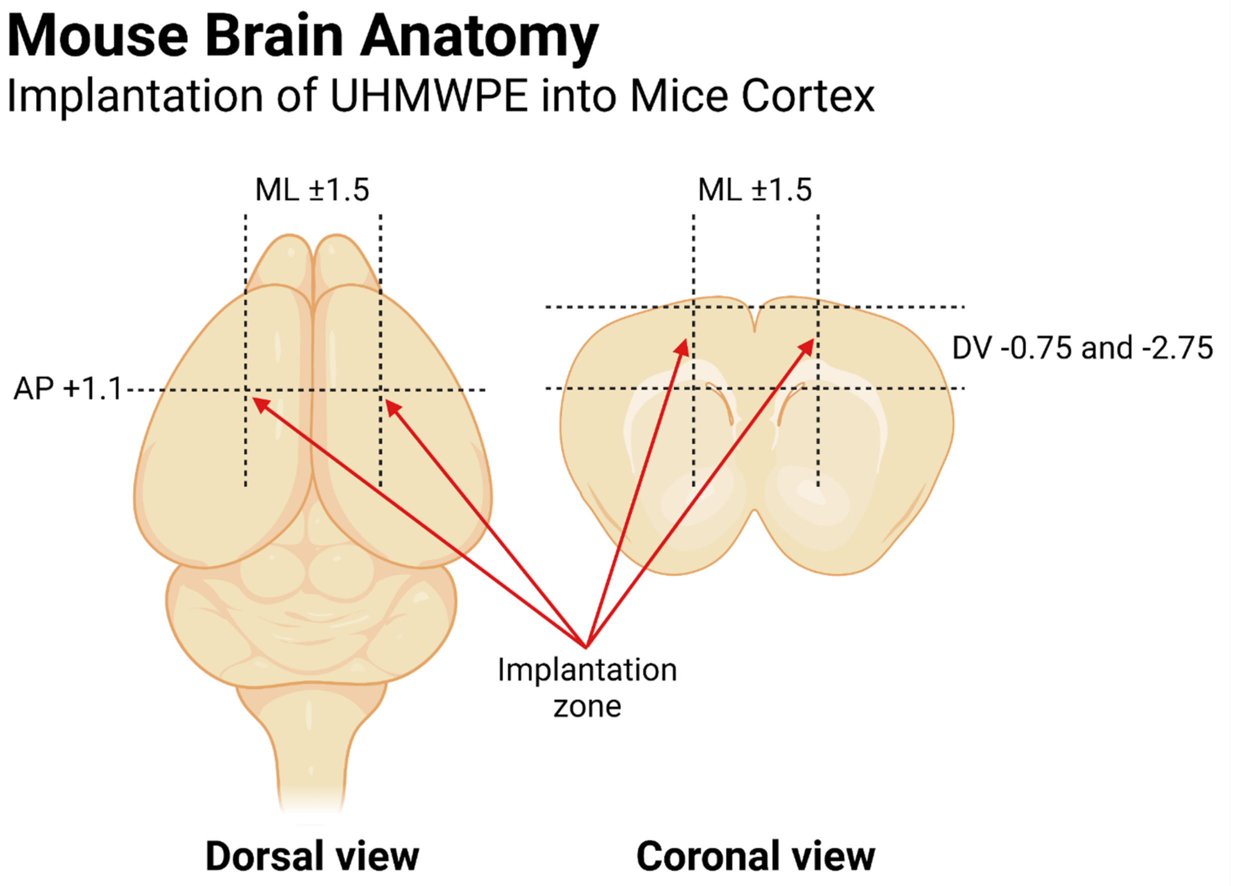

Supplemental Figure S2.
Illustration of UHMWPE implantation into mouse cortex. The stereotaxic coordinates were established according to the mouse brain atlas and UHMWPE slabs were implanted bilaterally into primary motor cortices (M1) and putamen (Pt) (AP: +1.1 mm anterior to bregma; ML: ±1.5 mm lateral to midline; DV: −0.75 and −2.75 mm depths from skull surface, respectively).
